# Williamson on Counterpossibles

**DOI:** 10.1007/s10992-017-9446-x

**Published:** 2017-08-16

**Authors:** Francesco Berto, Rohan French, Graham Priest, David Ripley

**Affiliations:** 1 Institute for Logic, Language and Computation (ILLC), University of Amsterdam, Amsterdam, Netherlands; 2 Department of Philosophy, School of Philosophical Historical and International Studies, Monash University, Melbourne, Australia; 3 CUNY Graduate Center, New York, USA; 4 Department of Philosophy, University of Connecticut, Storrs, CT USA

**Keywords:** Impossible worlds, Counterpossible conditionals, Nonvacuism, Counterfactual modal epistemology

## Abstract

A counterpossible conditional is a counterfactual with an impossible antecedent. Common sense delivers the view that some such conditionals are true, and some are false. In recent publications, Timothy Williamson has defended the view that all are true. In this paper we defend the common sense view against Williamson’s objections.

## Introduction

A counterpossible conditional is a counterfactual conditional with an impossible antecedent. According to some theorists, who we will call *vacuists*, all counterpossibles are true. According to others, who we will call *nonvacuists*, some counterpossibles are true, and some are false.^1^ In recent work, Williamson [48, 50] has taken up the cause of vacuism. The purpose of this paper is to evaluate Williamson’s arguments.

We will proceed as follows. In Section 2, we recall some motivations for both vacuism and nonvacuism, and sketch a sample nonvacuist semantics for counterfactuals using impossible worlds, to serve as a target for Williamson’s arguments. In Section 3, we present and rebut three arguments Williamson has given against nonvacuist semantics like the one we give. In Section 4, we present and rebut three attempts Williamson has made to undermine the intuitions that provide the most direct support for nonvacuism. In Section 5 we end by arguing that Williamson’s modal epistemology is not only compatible with nonvacuism, but actually leads in its direction.

## Vacuism and Non-vacuism

### The Consensus

We begin by considering the orthodox treatment of counterfactuals, inherited from Kratzer [21], Lewis [24] and Stalnaker [42].^2^ To evaluate a counterfactual conditional like

- If it hadn’t snowed last night, then John’s train wouldn’t have been late

we consider the closest^3^ possible worlds in which it didn’t snow last night, and see whether those are worlds in which John’s train isn’t late. A counterfactual  is true just in case all the closest *A*-worlds are *B*-worlds. Closeness is understood here as (largely contextually determined) similarity in the relevant respects, usually as minimal variation from the world of evaluation required to get the antecedent to come out true.^4^ The framework delivers the invalidity of certain (allegedly) intuitively invalid inferences involving counterfactuals, such as transitivity, contraposition, and antecedent strengthening.

It also delivers vacuism. If *A* is impossible, there are no *A*-worlds. Thus, for any *B*, *B* is, vacuously, true at all the closest *A*-worlds; the counterpossible  is true.

### Why Nonvacuism?

The issues we are to discuss arise when we consider conditionals like the following pair, essentially due to Nolan [30]:

1. If Hobbes had (secretly) squared the circle, all sick children in the mountains of South America at the time would have cared.
2. If Hobbes had (secretly) squared the circle, all sick children in the mountains of South America at the time would not have cared.

Squaring the circle is impossible. The set of possible worlds in which Hobbes (secretly) squares the circle is empty. As a result, on the orthodox account, both (1) and (2) are true.

This is a surprising result. It is intuitive, we take it, that (1) is false; it’s wrong to think that the children would have cared if Hobbes, *per impossibile*, had (secretly) squared the circle. Indeed, they wouldn’t even have known. But if (1) is false, then vacuism too is false. This has motivated the construction of nonvacuist semantic theories, which can deliver the intuitive verdict about (1).^5^

### How Nonvacuism?

One usual approach to nonvacuism (see for example [8, 10, 28, 30] amongst others) is to retain the contours of the orthodox account, while dropping the restriction to *possible* worlds. On such an approach, (1) can be false in the way any false counterfactual is: by having its consequent false at some of the closest worlds where its antecedent is true. Because it is impossible to square the circle, none of these worlds can be a possible world. So these approaches accept impossible worlds as well. In other respects, however, they match the orthodoxy.^6^

Here, we provide a simple nonvacuist semantic theory along these lines.^7^ We start with a propositional language with connectives  (the counterfactual conditional), and modal operators $\square $ and $\lozenge $. Let π be the set of propositional parameters, and let Φ be the set of formulas. An interpretation is a tuple $\left \langle W,P,\{R_{A}:A\in {\Phi }\},\nu \right \rangle $, where:

- *W* the set of worlds,
- *P* ⊆ *W* is the set of possible worlds, so *I* = *W* ∖ *P* is the set of impossible worlds,
- for every formula, *A* ∈ Φ, *R*_*A*_ ⊆ *W* × *W* is a binary relation on *W*,
- *ν* is a function which assigns the value 1 or 0 to every propositional parameter *p* ∈ π at every world; and to every formula *A* ∈ Φ at every *impossible* world. Write these as *ν*_*w*_(*p*) = 1 (or 0) and *ν*_*w*_(*A*) = 1 (or 0).

One may think of *w* *R*_*A*_ *w* ^′^ as meaning that *w* ^′^ is *ceteris paribus* the same as *w*, except that *A* holds there. If one likes talk of similarity, one can cash this out in terms of *w* ^′^ being a most similar world to *w* where *A* is true; and this will motivate certain constraints on *R*_*A*_. But similarity-talk is by no means unproblematic, and no means mandatory either.

Let us write $w\Vdash A$ to mean that *A* is true at *w*. The truth conditions of formulas at worlds *w* ∈ *I* are simple:

- $w\Vdash A$ iff *ν*_*w*_(*A*) = 1

Formulas are evaluated directly at impossible worlds. The truth conditions for worlds *w* ∈ *P* are the familiar ones:

- $w\Vdash p$ iff *ν*_*w*_(*p*) = 1
- $w\Vdash \lnot A$ iff it is not the case that $w\Vdash A$
- $w\Vdash A\wedge B$ iff $w\Vdash A$ and $w\Vdash B$
- $w\Vdash A\vee B$ iff $w\Vdash A$ or $w\Vdash B$
- $w\Vdash \Box A$ iff for all *w* ^′^ ∈ *P*, $w^{\prime }\Vdash A$
- $w\Vdash \Diamond A$ iff for some *w* ^′^ ∈ *P*, $w^{\prime }\Vdash A$
iff for all *w* ^′^ such that *w* *R*_*A*_ *w* ^′^, $w\Vdash B$

An inference is valid iff it is truth preserving at all *possible* worlds of all interpretations:

- Σ ⊧ *A* iff for every interpretation, and for every *w* ∈ *P*: if $w\Vdash B$ for all *B* ∈ Σ, then $w\Vdash A$

It is not difficult to see that at possible worlds the extensional connectives work classically, and the modal logic is standard S5.^8^

The only way in which these semantics differ from a standard semantics for counterfactuals is in the presence of impossible worlds; and these have an effect, note, only on counterfactuals. The impossible worlds are anarchic in the following sense: if Σ is any set of formulas, and *A* ∉ Σ, then there may be worlds in which *B* holds for all *B* ∈ Σ, but *A* does not. (Priest [36, Ch. 9] calls this the *Secondary Directive* of impossible worlds.)

We have described a basic system of conditional logic, where there are no constraints on the accessibility relations. (This is a conditional logic analogue of the basic modal logic, K.) Stronger systems can be obtained, as usual, by adding constraints on the *R*_*A*_s. The intended understanding of the accessibility relation motivates the following constraints:

- If *w* *R*_*A*_ *w* ^′^ then $w^{\prime }\Vdash A$
- If $w\Vdash A$ then *w* *R*_*A*_ *w*

The former says that *R*_*A*_-accessible worlds will be *A*-worlds—worlds making *A* true. The latter expresses the thought that if *A* is true at *w*, then *w* is one of the worlds that is *ceteris paribus* the same as *w* except that *A* holds. It corresponds to Lewis [24]’s “weak centering”. The conditions have an effect only when *w* is a possible world, since the *R*_*A*_s are not involved in determining the truth value of anything at an impossible world. As is not difficult to check, they guarantee, respectively, that  satisfies counterfactual self-implication and *modus ponens*:

These inferences seem obviously desirable for the counterfactual conditional.

A systematic discussion of what further constraints might be placed on the accessibility relations would be out of place here. However, one constraint will play an important role in what follows:

- If $x\Vdash A$ for some *x* ∈ *P*, and *w* *R*_*A*_ *w* ^′^, then *w* ^′^ ∈ *P*

If *A* is true at some possible world, and *w* *R*_*A*_ *w* ^′^, then *w* ^′^ is possible. (We may, in fact, restrict the condition to just possible *w* s, since, as noted, accessibility plays no role at impossible worlds.) To evaluate the truth at a possible world of a conditional with a possible antecedent, we do not look at impossible worlds. Thinking of the accessibility as expressing closeness between worlds, this amounts to the claim that for any possible world, *w*, any possible world is closer to *w* than any impossible world. One may therefore call this the ‘Strangeness of Impossibility Condition’, or SIC. Thinking of the accessibility relation in these terms makes the condition somewhat contentious.^9^ However, thinking of the relation as expressing a *ceteris paribus* condition can make the condition seem natural. If *A* is possibly true, then if *w* is a possible world, we should expect a world that is *ceteris paribus* the same as *w* except that *A* is true, to be itself possible. At any rate, as is easily checked, the constraint validates the inference:

which is a notational variant of the principle called POSSIBILITY in Williamson [48, p. 156]. SIC has further important consequences for validity, as we shall see.

It is a non-trivial matter to extend the semantics to a first-order language in such a way as to get the quantifiers to work properly. We need not go into details here, since they are not germane to what follows.^10^ However, identity will be relevant in the discussion to come, so something must be said about this. Suppose, then, that our language is augmented by the identity predicate, =, and a set of constants, in such a way that if *a* and *b* are any constants, *a* = *b* is an atomic formula. We may now handle the semantics of identity with constraints on interpretations. For possible worlds *w*, *w* ^′^, the constraints are the obvious ones:

- *ν*_*w*_(*a* = *a*) = 1
- if *ν*_*w*_(*a* = *b*) = 1 then *ν*_*w*_(*A*) = *ν*_*w*_(*A*_*b*_(*a*))

where *A* is any atomic sentence (note the restriction to *atomic* sentences here: it will matter) and *A*_*b*_(*a*) is *A* with any number of occurrences of *b* replaced by *a*. Truth values of identity statements are also invariant across possible worlds, so:

- $\nu _{w}(a=b)=\nu _{w^{\prime }}(a=b)$

It is now easy to establish that if *A* is any sentence in which *a* does not occur within the scope of a , and *w* is any possible world, then if $w\Vdash a=b$: $w\Vdash A$ iff $w\Vdash A_{b}(a)$. So the substitutivity of identicals (SI) holds in such contexts.

There are no constraints on atomic sentences at impossible worlds. It follows, in line with the Secondary Directive, that there may be such worlds, *w*, where it is not the case that $w\Vdash a=a$; and where $w\Vdash a=b$ and $w\Vdash Pa$, but it is not the case that $w\Vdash Pb$.^11^ It also follows, again in line with the Secondary Directive, that even if $w\Vdash a=b$ and *w* is a possible world, one may have an impossible world, *w* ^′^, where $w^{\prime }\Vdash Pa$, but it is not the case that $w^{\prime }\Vdash Pb$.^12^

It follows from the latter fact that SI is not valid when substitution is within the scope of counterfactuals. We will come back to this; note for now that such invalidity is to be expected when impossible antecedents are around. For example (see Priest [35, §19.5.4]):

- If the Morning Star were not the Evening Star, then modern astronomy would be badly mistaken.

But the Morning Star is the Evening Star, and it is not the case that:

- If the Morning Star were not the Morning Star, then modern astronomy would be badly mistaken.

Rather, it would be modern logic that is badly mistaken.

This framework gives a simple nonvacuist semantics. Consider an interpretation with just two worlds, @, which is possible, and *w*, which is not. Let *A* be any logical falsehood, let @ access *w* and only *w* under *R*_*A*_, *ν*_*w*_(*A*) = 1 and *ν*_*w*_(*B*) = 0. Then  is false at @.

With this in hand, let us move on to Williamson’s criticisms.

## Objections to Nonvacuism

We divide Williamson’s objections into two camps. In the present section, we consider objections directed at nonvacuist semantic theories like the one offered above. In Section 4, we consider objections to the intuitions that motivate nonvacuism in the first place.

### Weak Logic

Williamson [48, p. 174] offers the following objection: “We may also wonder what logic of counterfactuals [nonvacuists] envisage. If they reject elementary principles of the pure logic of counterfactual conditionals, that is an unattractive feature of their position”.

Williamson does not say which logic he has in mind as “the pure logic of counterfactual conditionals”, or which of its principles are “elementary”. However, Williamson [49, p. 85] makes use of a counterfactual logic; we assume that the distinctively counterfactual axioms and rules of this system might give some idea. We consider two:^13^

- **REFLEXIVITY:**
- **CLOSURE:** If $\vdash (B_{1} \land {\ldots } \land B_{n}) \supset C$ then

The principle REFLEXIVITY holds in the logic of Section 2.3. The principle CLOSURE does not. But should one expect this in a nonvacuist semantics? A particular case of this is: If $\vdash B \supset C$ then . But classically, $\vdash (p \land \neg p) \supset (q \land \neg q)$. So, . Now take *p*∧¬*p* for *A*. Then using REFLEXIVITY and detaching, we get . That is, such a logic requires that any contradiction counterfactually implies any other. Nonvacuists, of course, think it is an attractive aspect of their view that it allows us to reject such a conclusion. It’s wrong to think that if 2 were both equal and not equal to 3 then it would be raining and not raining. But to maintain this, one of REFLEXIVITY, CLOSURE, or classical logic must give. In the logic of Section 2.3, it is CLOSURE.

CLOSURE, then, may be a basic rule of the logic Williamson has in mind for counterfactuals, but it should not be accepted. Counterfactual suppositions can take us beyond logical bounds; they can lead us to entertain situations in which logically equivalent claims come apart, or in which a claim can hold without all its consequences holding. For vacuists, these are not ‘unattractive features’ of their view: they provide one of the main intuitive motivations for it.^14^ (Of course, such intuitions can be challenged; that is the topic of Section 4.)

While CLOSURE should be rejected by nonvacuists, there is a closely related principle that they may accept. This is:

- **P-CLOSURE:** If $\vdash (B_{1} \land {\ldots } \land B_{n}) \supset C$ then

P-CLOSURE is just like CLOSURE except that the validity it yields has as a premise that a certain claim is possible. Because the logic of Section 2.3 does not validate $\lozenge (p \land \neg p)$, the argument above against CLOSURE does not extend to P-CLOSURE .

P-CLOSURE holds of the logic we have specified, if we assume SIC. And quite generally, with SIC in place, as long as the antecedents of all the conditionals we are dealing with are possible, we can simply ignore the impossible worlds. So the valid inferences of merely-possible-world semantics are recoverable enthymematically by adding suppressed premises of the form ♢*A*. Adding impossible worlds loses us nothing.

The logic of counterfactuals we give is perhaps weaker than Williamson would like. But this is not an ‘unattractive feature’; rather, it’s what’s required to capture the intuitions the nonvacuist begins from.^15^

### Substitution of Identicals

Consider the following pair (numbers as in Williamson [48], pp. 174–6):

(32) If Hesperus had not been Phosphorus, Phosphorus would not have been Phosphorus.
(33) If Hesperus had not been Phosphorus, Hesperus would not have been Phosphorus.

We take the appropriate evaluation of these to be as follows: (32) is false, (33) is true. (33) is an instance of REFLEXIVITY, which we endorse. (32), on the other hand, is implausible; although Hesperus and Phosphorus *are* identical, if they *had not been* nothing follows about the self-distinctness of one of them. In particular, there is no reason to expect Phosphorus to be self-distinct in such a scenario. The semantics we have presented gets this right: *a* = *b* does not entail . Substitutivity of identicals can fail on this semantics when (and only when) the substitution in question is within the scope of a counterfactual.

That is, we take it that counterfactuals create *hyperintensional* contexts, contexts in which substitutivity of identicals is not valid. This is not required by nonvacuism per se; but it is supported by the very same kinds of intuitions that support nonvacuism.

Williamson [48, p. 175], however, holds that this is “highly implausible”. The reason given there has two premises: that hyperintensionality occurs only in constructions that are “about representational features” (that is, constructions that are broadly epistemic or intentional, like ‘It is a priori …’ or ‘Alice believes …’); and that counterfactuals are not about representational features in this way.

We grant arguendo that counterfactuals are not broadly epistemic or intentional,^16^ to focus on Williamson’s other premise. An operator or a context’s being hyperintensional simply does not imply its being representational or broadly epistemic. There are hyperintensional contexts that are not in any way ‘about representational features’ (see Nolan [31]), and counterfactuals may well be among these. Hyperintensionality without appeal to representation is invoked in many discussions of metaphysical grounding; see for example papers in Correia and Schnieder [11], like Fine [13] and Koslicki [19]. On connections between grounding and counterfactuals, see Krakauer [40] and Schaffer [20]. Wilson [51] argues that nonvacuism follows from a counterfactual approach to grounding. The claim that hyperintensionality as such requires being about representational features would need serious support; and this Williamson does not offer.

To see how counterfactuals might be hyperintensional without being about representations, simply return to the semantics of Section 2.3. We can assume, together with (Kripke [22]; Marcus [26]; Williamson [48, p. 161]), that if *a* = *b*, then it is necessary for *a* to be *b*. Notice that our semantics above conforms to this: the truth values of identity statements ‘*a* = *b*’ do not change across possible worlds. Then *a*’s not being *b* is a way things just cannot be: an impossibility. In particular, it can be so at an impossible world. There is nothing particularly epistemic about this, any more than there is about a world which hosts a physical impossibility, such as (supposing Einstein was right) something accelerating through the speed of light. A world is partially characterised by a set of sentences. These tell you exactly what the world is like—whether it is possible or impossible. And if it be retorted that if *a* = *b*, and this statement really is about *a* and *b*, the failure of substitutivity would be impossible, the reply is ‘Of course’!

So Williamson’s argument about ‘representational features’ fails. But one might still think that counterfactuals allow for substitution of identicals. Williamson [48, p. 174] tries to bolster this impression with the following argument (numbered as there):

(34) If the rocket had continued on its course, it would have hit Hesperus.
(35) Hesperus = Phosphorus
(36) If the rocket had continued on its course, it would have hit Phosphorus.

The argument from (34) and (35) to (36) is, Williamson claims, “unproblematically valid”.

But the argument is not valid: it turns on a step of substitution within a counterfactual conditional, which we have seen is not in general truth-preserving. (This particular argument *is* truth-preserving, but a truth-preserving instance of an argument form lends no support to the claim that the form itself is valid.) However, we have allowed for substitution at *possible* worlds. So in the present example, the substititution could not go wrong if there were some guarantee that we remained within the possible. Given SIC, this allows us to see this argument as enythmematic, with missing premise ♢(The rocket continued on its course).

### Reductio Arguments

Another Williamsonian objection to nonvacuism, found in both Williamson [48, 50], comes from reductio arguments.^17^ Reductio arguments are, of course, crucial to mathematics as it is practiced. Williamson attempts to show that nonvacuists about counterpossibles must hold current standard mathematical practice to be mistaken.

Although Williamson admits that mathematical practice does not *depend* on using counterfactuals in the formulation of reductio arguments, he calls it “surely legitimate” to do so. And indeed, there is some temptation to assert counterfactuals when reporting a particular line of reasoning by reductio, and also when explaining what it is that makes reductio reasoning valid in the first place: ‘It can’t be that *A*, because if it were that *A*, then it would be that *B*; but *B* is wrong, so *A* too must be’. This kind of reasoning is perfectly valid in the semantics we have presented; we have . This comes from the classicality of the base world plus weak centering; nothing more is required. The presence of impossible worlds provides only extra ways for  to *fail*, and so does nothing to affect this argument: it is valid for the vacuist and nonvacuist alike.

The trouble stems from certain counterpossibles that are or can be used in reductio reasoning in this way. Since the reasoning is good, the counterpossibles ought to come out true. However, Williamson claims that nonvacuists cannot make good on this prediction, and end up calling the counterpossibles false.

Williamson [50] considers the following examples:^18^

(56) If there were a largest prime *p*, *p*! + 1 would be prime.
(57) If there were a largest prime *p*, *p*! + 1 would be composite.
(58) If there were a largest prime *p*, *p*! + 1 would be both prime and composite.

Williamson considers the following proof that there is no largest prime: first, show (56) and (57) on their own merits. Then, conclude (58) from them. Finally, appeal to our knowledge that no number is both prime and composite to conclude that there is no largest prime.^19^ Again, the final step of this reasoning is unproblematic for vacuists and nonvacuists alike; the alleged trouble for the nonvacuist is in getting (56)–(58) to come out true.

Why is (56) meant to be true? Because *p*! + 1 is not divisible by *n* for any *n* ≤ *p*, and if *p* is the largest prime all primes must be ≤ *p*. So *p*! + 1 has no prime factors at all, and so none other than itself; it must therefore be prime. Why is (57) meant to be true? Because *p*! + 1 is greater than *p*, and if *p* is the greatest prime everything greater must be composite. And why is (58) meant to be true? Because (56) and (57) are, and they have the same antecedent, so we can conjoin their consequents.

But by what right, Williamson objects, can the nonvacuist endorse these claims? If there *really were* a largest prime *p*, after all, the natural numbers would be very different from how they in fact are. So why should one expect the given reasoning to work even in such an impossible situation?

One way to answer this objection is simply to claim that counterfactuals are not really being used in the proof at all. A counterfactual of the form  is just a *façon de parler* for the thought that *B* follows from the assumption that *A*. The role of counterfactual talk is then merely to signal *A*’s role as an assumption (to be later discharged) in the reasoning to follow.^20^

However, a different reply is illuminating for other reasons. Let us consider the role of context in counterfactuals.^21^ Any broadly Kratzer-, Lewis-, or Stalnaker-like approach to counterfactuals involves two key ingredients: an underlying space of worlds or situations, and some apparatus for focusing on the ones relevant to interpreting the counterfactual at hand. *All* existing approaches to counterfactuals, vacuist and nonvacuist alike, take the second ingredient to be sensitive to the context in which a counterfactual occurs: there is simply no other way to get sensible results. As Lewis [24, p. 92] puts the point, “The truth conditions for counterfactuals … are a highly volatile matter, varying with every shift of context and interest”.^22^

This contextual variation is effected in different ways in different formalisms. In the semantics of Section 2.3, it is linked to the *R*_*A*_ relations; different contexts will determine different *R*_*A*_s. When we consider what would be the case if *A* were the case, the collection of worlds accessed is determined not by *A* alone, but by the interaction of *A* and the context of assertion. Context determines which aspects of reality we attempt to hold fixed as we modify things to make room for the truth of the antecedent.^23^

In contexts where (56)–(58) are uttered in the course of the imagined reductio argument, they are true. Conversational participants hold fixed what they know about the additive and multiplicative structure of the natural numbers; with such facts fixed, the claims follow easily, for the reasons sketched above. In the context of a mathematical proof in standard arithmetic, facts about addition, division, etc. are *exactly* the kind of things held fixed. What else would one expect?

This does not mean, however, that such mathematical facts must be held fixed in *every* conversational context. Thus, for example, in a discussion of mathematical finitism,^24^ it could be quite correct to say that if there were a greatest number, there would be a greatest prime number. In such a context, we would not hold fixed that every number has a successor. Or we might discuss what the physical world would be like if there were a largest prime number. Again, we cannot allow all of the facts of standard arithmetic to carry over.^25^

We conclude that nonvacuist approaches such as the one presented above do not impose a problematically weak logic; that there is no trouble in failing to allow for substitution of identicals within counterfactuals; and that nonvacuists can make good sense of counterfactuals that seem to play a role in mathematical reasoning.

## Questioning Nonvacuist Intuitions

A main motivation *for* nonvacuism remains intuitive.^26^ Williamson has taken up another line of attack against nonvacuism, centering on such intuitive support. He grants that the relevant intuitions are present, but argues that they are not veridical. We consider three Williamsonian arguments in this ballpark.

### Thinking it Through

The first concerns an example due to Nolan [30]. (See also the discussion in Brogaard and Salerno [9]). Suppose that I am asked ‘What is 5 + 7?’, and answer ‘11’. Consider the following sentences (numbering from Williamson [48, p. 172]’s discussion of this case):

(30) If 5 + 7 were 13 I would have got that sum right.
(31) If 5 + 7 were 13 I would have got that sum wrong.

At first blush, (30) seems false and (31) true. Of course, if (30) is actually false then so is vacuism, so long as it is necessary that 5 + 7 isn’t 13. Williamson responds to this case:

[Such examples] tend to fall apart when thought through. For example, if 5 + 7 were 13 then 5 + 6 would be 12, and so (by another eleven steps) 0would be 1, so if the number of right answers Igave were 0, the number of right answers Igave would be 1. We prefer (31) to (30) because the argument for (31) is more obvious, but the argument for (30) is equally strong. (p. 172)

It seems to us, though, that the argument for (30) is not equally strong. To see this it suffices, again, to note the role of context.^27^ As we pointed out in Section 3.3, whether a particular chain of reasoning succeeds or fails in supporting the truth of a counterfactual depends on the context, and in particular what truths about the case need to be held fixed to legitimate the reasoning.

In this case, all we need to hold fixed for (31) to be true is that the questioner asked what 5 + 7 is, that the answer given was 11, and that 11 is not 13. Williamson’s argument for (30) needs to hold fixed all of those same facts, plus facts about decrementing left and right addends (in particular, that 5 + 7 = 13 ⊩ 5 + 6 = 12—and its subtraction-generated cousins—are true),^28^ plus facts connecting ‘number of right answers’ given to whether someone gets an answer right.^29^

The contexts in which (30) comes out true, then, are a superset of those in which (31) comes out true. So long as there are contexts in which we can let facts about decrementing and incrementing vary, for example, it is a proper superset. But to suppose that 5 + 7 is 13 is to suppose that the additive structure of the numbers is something other than it actually is. Without some special context (like, say, being in the course of a certain kind of mathematical proof—again, compare Section 3.3), we have reason to expect that we should not hold fixed facts about incrementing and decrementing under such a supposition. So without some special context, we should expect that (31) is true and (30) not. In other words, for Williamson’s argument to work, he needs to argue that we are in such a context, which he does not attempt.

Williamson is not sensitive to this point, we suspect, because he is refusing to allow any necessary facts to vary at all under counterfactual supposition, regardless of context. It is as though he treats every context as if it were one of mathematical proof. But this refusal is dialectically inappropriate; it amounts to assuming what is at issue. Insisting on holding all necessary truths fixed will undermine one’s ability to reach intuitive verdicts about counterpossibles—and this has been clear from the get-go. Thinking things through, then, undermines Williamson’s argument, not Nolan’s example.

### A Heuristic?

Williamson’s main attempt to undermine nonvacuist intuitions works by proposing a particular hypothesis about how these are reached. Recall (1):

1. If Hobbes had (secretly) squared the circle, all sick children in the mountains of South America at the time would have cared.

No matter how we come at this sentence, we find it stubbornly seeming to be false.

Here is Williamson [50]’s explanation for this seeming. We naturally take counterfactuals of the form  and  to be contraries: ‘If you were to win the lottery you would be happy’ and ‘If you were to win the lottery you would not be happy’ cannot both be true.^30^ According to Williamson, this natural tendency is the result of a fallible heuristic, (HCC*).^31^

- (HCC*) If you accept one of  and , reject the other.

According to Williamson, then, we take a counterpossible  to be false because we have computed the truth value of , found it to be true, and applied (HCC*) in order to conclude that  is false.

On its face, this theory is worryingly ad hoc: the only evidence we have for the presence of a heuristic like (HCC*) is the very intuitions, inconvenient for vacuism, it is invoked to explain away. But it has two additional problems. First, there seem to be a range of cases in which intuitive judgments do not accord with what (HCC*) would predict. Second, the picture Williamson offers of how (HCC*) enters into our intuitive judgments seems implausible.

If it is (HCC*), rather than semantic competence, that explains speakers’ judgments that some counterpossibles are false, two things follow. Faced with counterpossibles  and , speakers should not judge both to be true, and speakers should not judge both to be false. They should not judge both to be true because (HCC*) militates against it: having judged one of the two to be true, a speaker making use of (HCC*) should thereby judge the other to be false. And they should not judge both to be false because these judgments would be inexplicable: to get a verdict of falsity for one of these conditionals from (HCC*), the other one must have been judged true.

But there are cases in which counterpossibles  and  both seem true, and cases in which both seem false. For example, both of

- If it were raining and not raining, it would be raining
- If it were raining and not raining, it would not be raining

appear to be true. And both of

- If it were raining and not raining, it would be Tuesday
- If it were raining and not raining, it would not be Tuesday

appear to be false. Williamson’s theory cannot explain these intuitions. Williamson might simply deny them. But if not, the former pair shows that (HCC*) is not used generally, even where it could easily apply; and the latter pair shows that at least some cases of counterpossibles being judged false cannot be explained by (HCC*).

To show the implausibility of Williamson’s account of the application of (HCC*), we begin from his own general picture of counterfactual judgments, Williamson [48, p. 147ff], which we find, on the contrary, quite plausible. On this picture, to evaluate a counterfactual , we imagine situations in which the antecedent holds, and check these imaginations to see whether the consequent holds robustly in such situations. But there is no place in this process for (HCC*) to act. So Williamson wants us to imagine that in the case of certain counterfactuals like (1), we don’t use this process. Rather, in such cases, Williamson has it, we shift our attention to the distinct sentence (2), and then evaluate (2) according to the usual procedure. Having judged (correctly) that (2) is true via this procedure, Williamson supposes that we then apply (HCC*) to reach the verdict that (1) is false. By supposing that speakers judging (1) first judge (2) in the ordinary way, Williamson opens up room for (HCC*) to apply.

We see no reason to resort to this more complex procedure. The general method will do; we may evaluate the truth of (1) directly in the usual ways. We just consider situations in which Hobbes squared the circle; and we see that the consequent does not generally hold in these. Hence we take the conditional to be false. (As an analogy, if one is trying to test whether *B* follows from *A* in some natural deduction system or sequent calculus, one does not first have to test whether $\lnot B$ does.) Finally, we note, the claim that we evaluate (2) before (1) seems entirely ad hoc. We could just as well have started by evaluating (1), so reversing the picture.

Williamson’s proposed heuristic, then, makes mistaken predictions about speaker judgments of counterpossibles. It requires counterpossibles like (1) to be judged in a different way from counterfactuals generally (and from counterpossibles like (2)) – a way that is both more complex and unmotivated.

### Vacuous Quantification

Williamson [48, p. 173] also makes an attempt to undermine trust in nonvacuist intuitions based on an analogy with vacuous (universal) quantification. “The logically unsophisticated”, he has it, find it intuitive that, given that ‘Every golden mountain is a mountain’ is true, then ‘Every golden mountain is a valley’ should be false, for being a mountain and being a valley are incompatible. However, both claims are true, vacuously, if there are no golden mountains. People extrapolate wrongly from familiar cases, in which one does not quantify vacuously.

The point is expanded in Williamson [50, §6]. A natural inclination, we are told, is to judge:

- Every dolphin in Oxford has arms and legs
- Every unicorn is hornless

as false, even though there no unicorns or dolphins in Oxford. Despite this inclination, these claims are true, because there are no unicorns or dolphins in Oxford.

The intended analogy with vacuous quantification is clear: if there are no circumstances in which the antecedent of a counterfactual is true, then counterfactuals with that antecedent are true. This is because for a counterfactual to be untrue there must be circumstances at which its antecedent is true. But the analogy looks question-begging: what is at issue is whether there are such circumstances, not what would happen in their absence.

We note, also, that Williamson has an objectionable view of the history of universal quantification. He says (§6):

Our theoretical grasp of universal quantification is currently more secure than it is of counterfactual conditionals [...]. But it was not always so. Centuries of confusion about the existential import or otherwise of the universal quantifier bear witness to the difficulty of achieving aclear view or the truth conditions of sentences of our native language formed using the most basic logical constants.

But the consensus of the great medieval logicians, including Scotus, Ockham, and Buridan, was that all of these sentences are false, since ‘Every *P* is *Q*’ entails ‘Some *P* is *Q*’. These thinkers had perfectly precise theories of restricted quantification, consistent with both Aristotelian syllogistic and the Aristotelian square of opposition (see Read [37]). In disagreement with contemporary logic they may have been; confused they were not.^32^

Finally, we note that Williamson means to extend his heuristics to these vacuous quantifications. He proposes (§6) that we judge universal quantifications according to the heuristic he calls (HUQ*): “If you accept one of ‘Every *σ* *ϕ*s’ and ‘Every $\sigma \lnot \phi $s, reject the other”. This, he takes it, explains the intuition that some such vacuous quantifications are false. But this has the same problems as his (HCC*) hypothesis. In particular, it cannot explain the judgments of the medieval greats; they took *all* of these sentences to be false, and so could not have arrived at these judgments by applying (HUQ*).

## From Williamson’s Modal Epistemology to Nonvacuism

We end our discussion by considering the role vacuism plays in Williamson’s epistemology, and arguing that nonvacuism can be directly motivated on the basis of such epistemology.

### Counterfactual Paths to Necessity

Behind Williamson’s attacks on nonvacuism, we suspect, is the thought that vacuism is required to ground our knowledge of metaphysical modality in our assessments of counterfactual conditionals. This idea is at the core of Williamson [48]’s modal epistemology. The strategy is to construe claims of metaphysical necessity as equivalent to certain counterfactual claims, and argue on this basis that the epistemology of metaphysical modality reduces to the epistemology of counterfactuals.^33^

We won’t address here the extent to which Williamson’s reduction is successful (for some criticism, see Jenkins [16] and Peacocke [32]). We’ll just focus on the extent to which this project is tied to vacuism. If vacuism *is* required for it to work, that in itself may count somewhat in favour of vacuism. But Williamson’s approach is compatible with a certain kind of nonvacuism.

To characterize metaphysical necessity in terms of the counterfactual conditional, Williamson [48, 49], drawing on Lewis [24, pp. 21–24], present three candidate logical equivalents of $\Box A$:

The metaphysically necessary can be recognized as (a) that whose negation counterfactually implies *falsum* (notice that for Williamson ‘⊥’ is a placeholder for a contradiction Williamson [48, p. 156]); (b) what is counterfactually implied by its own negation; (c) what would be the case, whatever were the case. In the presence of vacuism, the first two are equivalent and, if one allows oneself an obvious set-up for propositional quantification, the third one is, too (Williamson [48, p. 297]). However, on a nonvacuist approach these are not equivalent, as we will see.

Given its simplicity, Williamson [48, p. 157] initially characterises $\Box A$ in terms of (a) above. In our setting we will enrich our language from Section 2.3 with the constant ⊥. We will assume the following semantics for ⊥: it is true at no possible world, and at all impossible worlds. (This is a violation of the Secondary Directive, but it seems harmless if ⊥ simply expresses the fact that we are in an impossible world—of course, $\lnot \bot $ can be true there too.)

With these assumptions in play, $\square A$ is indeed equivalent to : if one of them is true at a possible world, then so is the other; this suffices to show  and . First, suppose that $\square A$ is true at a possible world *w*. Then for every possible world *w* ^′^, *A* is true at *w* ^′^. Hence, $\lnot A$ is false at *w* ^′^. Now consider the value of  at *w*. We consider worlds, *w* ^′^, where $wR_{\lnot A}w^{\prime }$. Since $\lnot A$ is true at these, they must be impossible. So ⊥ is true at all of these; thus  is true at *w*. Conversely, suppose that  is true at a possible world *w*. Then every world *w* ^′^ with $wR_{\lnot A}w^{\prime }$ is such that ⊥ is true at *w* ^′^; that is, no such *w* ^′^ is possible. By SIC, then, it must be that ¬*A* is true at no possible world. This is to say that *A* is true at every possible world, and so $\Box A$ is as well.

We note, however, that (b) and (c) are quite different. In this setting, for a given sentence *A*, (c) entails (b), which in turn entails (a);^34^ but neither entailment is reversible.^35^ Thus, (b) and (c) are too strong to serve as equivalents for $\square A$. Only (a) will do.

Williamson’s official story from Williamson [48, pp. 141-65], concerning the cognitive mechanisms involved in our evaluation of counterfactuals and how they ground our assessment of claims of metaphysical necessity, is put largely in terms of (a). The equivalence between (a) and $\Box A$ can be captured on the present nonvacuist approach, given SIC and the assumption about ⊥. It is only the equivalence between (a)–(c) that fails on this nonvacuist picture, not the equivalence between (a) and $\square A$.

Recall that Williamson’s aim is to ground our knowledge of metaphysically modal truths in our knowledge of certain counterfactuals. One might worry that this project is put in jeopardy by the switch from Williamson’s ⊥, which is $p \land \lnot p$, for some *p*, to the approach we have given to ⊥, on which it holds in all and only impossible worlds. This latter ⊥ is clearly modal. So, on the nonvacuist replacement account we are offering, our modal knowledge would seem to presuppose a certain amount of modal knowledge.

Williamson’s original approach, however, is in the same boat. Although his characterization of ⊥ is not immediately modal, for it to work to yield *knowledge* that *A* is metaphysically necessary it is not good enough that ⊥ is not true, or even that it is known not to be true. For $\square A$ to be known modulo the recognition of the equivalence with , we need to know that ⊥ *cannot* be true. Substantial modal knowledge is still being presupposed. Our suggested approach makes this presupposed knowledge easy to come by—indeed, trivial: ⊥ is characterized in such a way that it is impossible for it to be true. Williamson’s modal epistemology, then, does not require vacuism.

### Supposing the Impossible

Indeed, Williamson’s modal epistemology may itself provide reasons to believe in impossible worlds, and so open adoor to nonvacuism. This is so because of akey claim in the Williamsonian account of Williamson [48, Chapter 5]: that we can suppose and, perhaps, imagine absolute impossibilities.^36^ Williamson claims that knowledge of necessity can be had by coming to know certain counterfactuals. Knowledge of these, in turn, is obtained as follows:

One supposes the antecedent and develops the supposition, adding further judgments within the supposition by reasoning, offline predictive mechanisms, and other offline judgments. [...] To afirst approximation: one asserts the counterfactual conditional if and only if the development eventually leads one to add the consequent. (Williamson [48, p. 152–153])

In particular:

We assert $\Box A$ when our counterfactual development of the supposition that $\lnot A$ robustly yields acontradiction. (Williamson [48, p. 163])

But this means that, if $\Box A$ is correct, we have been mentally representing an impossibility. Williamson does not provide a semantics for supposing or imagining. But in supposing, or imagining, an impossible situation, are we not considering (not in all detail) an impossible world, or scenario? A natural semantics for ‘Cognitive agent *x* Ψ’s that *A*’, ‘Ψ’ standing for the relevant intentional state, is one in which the operator is understood in terms of (restricted) quantification over worlds, mimicking the ordinary possible worlds semantics for intentional (epistemic and doxastic) operators. (See, for example (Berto [7]; Goddard and Routley [14, §7.2.8]; Priest [36, Ch. 9].) And since we can represent impossibilities (as Willamson agrees), the worlds in question must at least sometimes be impossible. So Williamson’s epistemic project is not only compatible with nonvacuism, it naturally leads to impossible worlds on its own.

## Conclusion

Williamson’s arguments should not worry nonvacuists. His theoretical arguments do not reveal troubles in nonvacuist semantic theories, and his attempts to undermine nonvacuist intuitions are unconvincing. Finally, vacuism may not be required for a Williamsonian approach to modal epistemology. Indeed, the impossible worlds often invoked by nonvacuists may play a natural role in it.

## Footnotes

1 This leaves out an option: that they are all false. We will not consider this possibility here, but see Kment [18] for discussion.

2 Precursors can be found in Sprigge [41] and Todd [44]. The debate between vacuists and nonvacuists has been with us from the beginnings of this orthodoxy; see for example Goddard and Routley [14, p. 454]’s nonvacuist criticism of Montague [29]’s vacuist treatment of conditionals.

3 This relies on what Lewis [24, pp. 19–20] calls the Limit Assumption: for no world do we have worlds that get closer and closer to it, endlessly. Nothing in the opposition between vacuism and nonvacuism hinges on this.

4 Which aspects of similarity need to count as relevant in order to deliver the intuitively good results is a complicated business: see e.g. chapters 12 and 13 of Bennett [4].

5 Some vacuists, e.g., [24, p. 25], have denied that there are any such intuitions of falsity. But all the arguments we consider here are compatible with the existence of these intuitions. (Of course, vacuists must hold that these intuitions are *mistaken*, but this is different from denying their existence.)

6 Counterfactual conditionals are hardly the only place where impossible worlds come in handy. Impossible worlds are also helpful in dealing with puzzles concerning content [14, 15] and intentionality [36, 39] generally. Just as with possible worlds, there are a range of views as to the nature and metaphysical status of impossible worlds. We do not enter into this debate here, but see [3, 6, 8, 15, 52].

7 The following draws on Priest [35, Ch. 5].

8 The logic also obeys standard structural rules; we will take these entirely for granted in what follows.

9 It is rejected in Nolan [30], where it receives this name, and in Bernstein [5] and Vander Laan [46]; but it is accepted in Jago [15] and Mares [27].

10 The semantics for quantified conditional logics can be found in Priest [35, Ch. 19]. However, these have only possible worlds. The semantics with impossible worlds needs to employ the matrix semantics of Ch. 18, as deployed in Ch. 23. See also Priest [36, Ch. 1].

11 In the full semantics, this is achieved by letting the extension of the matrix *v*_0_ = *v*_1_ be arbitrary. See Priest [35, §23.6].

12 That is, this semantics violates the condition called ‘weak matching’ in [12]. See Ripley [38] for further discussion of this condition. Again, in the full semantics, the matrix technique is needed. See (Priest [35, Ch. 17]; Priest [36, Ch. 2]).

13 We need not discuss the principle Williamson calls VACUITY, which is motivated directly by appeal to vacuism; and the considerations concerning the principle Williamson calls EQUIVALENCE are much the same as those concerning CLOSURE.

14 Williamson [50] opens with three quick arguments for vacuism. Although we do not discuss them here, they fit in the present section; they assume principles that, like CLOSURE , are obviously incompatible with the intuitions that motivate nonvacuism in the first place.

15 For more discussion of principles like CLOSURE and their relation to nonvacuism, see Pollock [33], Wierenga [47] and Zagzebski [53]. One might also consider different variants of these principles, such as the following:

- If *B*_1_, …, *B*_*n*_⊢^⋆^ *C*, then

Here, ⊢^⋆^ might be a consequence relation *different* from the target ⊢. These principles would be satisfied on the kind of approach we have presented if the impossible worlds themselves are forced to obey the logic ⊢^⋆^. For more about this kind of approach, see (Bjerring [8]; Priest [36, §1.7]).

16 But: Lycan [25] takes conditionals to be epistemically flavoured, for reasons that have nothing to do with nonvacuism. Relatedly, Thomasson [43] takes metaphysical modality itself to be partly representational. And Brogaard and Salerno [10, p. 654] appeals directly to an alleged epistemic aspect of counterfactuals to explain failures of substitutivity like the one considered here. These are issues for Williamson, not for us.

17 Williamson [48, p. 174] claims that “some objectors” (that is, nonvacuists) have pointed to reductio arguments in support of nonvacuism, but the only reference in the surroundings is to Nolan [30], which is explicit in rejecting any such support. Similarly, the primary target of Williamson [50] is Brogaard and Salerno [10], which does not discuss reductio arguments. The other nonvacuists cited there are Nolan [30] (again) and Kment [18], which also does not discuss reductio arguments. For what it’s worth, our own past endorsements of nonvacuism also do not claim such support. We do not know who Williamson has in mind.

18 (56) and (57) come from Lewis [24, p. 25].

19 This is not the *usual* proof that there is no largest prime. (This shows that for any prime, *p*, there must be a larger. Either *p*! + 1 is prime or, if not, there must be a prime between *p* and *p*! + 1.) But it is a fine one for our purposes.

20 Or perhaps, as an anonymous referee suggests, we could simply reject counterfactual talk entirely, and insist on indicative versions of (56)–(58).

21 For more detailed consideration of the roles context can play in the interpretation of counterpossibles, see Vander Laan [46].

22 Of course this is not to say (and Lewis certainly does not think, as he goes on to make clear) that anything goes; although some of *us* may believe this, nothing so extreme is needed for the present response. We mean to point to a particularly modest instance of context sensitivity.

23 For a familiar example, consider this pair of counterfactuals, discussed in Lewis [24, pp. 66f.]:

- If Caesar had been in command [in Korea] he would have used the atom bomb.
- If Caesar had been in command [in Korea] he would have used catapults.

There are contexts in which the first seems true and the second false, and contexts in which just the opposite is the case. The first kind of context is one in which we hold fixed the technology available in the Korean War, and Caesar’s overall style of approach. The second kind of context is one in which we hold fixed the kind of army Caesar had at his disposal.

24 As in van Bendegem [45].

25 For examples of contexts like the latter, see Baron et al. [2].

26 We note that there are others. See Jenny [30] and Nolan [17].

27 We remark that at the final stage of the argument for (30), Williamson substitutes 1 for 0, which, as explained, we take to be invalid.

28 There are inconsistent arithmetics where one can have *n* + 1 = *m* + 1 without having *n* = *m* (even though $n+1=m+1\supset n=m$. $\supset $ does not detach; see Priest [34, Ch. 17].)

29 That the argument needs to hold fixed that 11 is not 13 is perhaps not obvious, so we pause here to explain. Note that the argument needs to conclude that the number of right answers given was 0 to proceed to the claim that it is 1. The only way we see to reach the conclusion that the number of right answers given was 0 is to appeal to 11’s distinctness from 13.

30 Indeed, Williamson suggests that we may confuse  with , thus taking them to be contradictories. But whether or not this is so, contrariety is all his explanation requires.

31 Williamson discusses two potential heuristics, (HCC) and (HCC*). We work with (HCC*) because it is the one Williamson prefers; but the two provide, essentially, the same explanation for the key intuitions, and they are subject to the same objections.

32 Williamson [50, §6], says “In the case of the universal quantifier, a clear understanding was finally achieved though systematic, highly general semantics and logical theorising, rather than a more data driven approach”. This mischaracterizes the great medieval logicians, whose work *was* highly systematic logical theorising.

33 See also Kroedel [23].

34 That (c) entails (b) is straightforward: if everything counterfactually implies *A*, then $\lnot A$ does. That (b) entails (a) is also fairly easy to see. First, notice that if  is true at a possible world *w*, it must be that *A* is true at each *w* ^′^ such that *w* *R*_¬*A*_ *w* ^′^. But then each such *w* ^′^ must be impossible (since ¬*A* is true at all of them as well), and so ⊥ is true at each of them.

35 Consider a model 〈*W*, *P*, {*R*_*A*_ : *A* ∈ Φ}, *ν*〉 with:

- *W* = {*w*_1_, *w*_2_, *i*_1_, *i*_2_}
- *P* = {*w*_1_, *w*_2_}
- *w*_1_ *R*_¬*p*_ *i*_1_, *w*_2_ *R*_¬*p*_ *i*_2_, *w*_2_ *R*_*q*_ *i*_1_
- *p* is true at *w*_1_, *w*_2_, and *i*_2_; ¬*p* is true at *i*_1_ and *i*_2_; *q* is true at *i*_1_ only.

In this model,  is true at *w*_1_ (and *w*_2_); but  is not true at *w*_1_, showing that (a) does not entail (b). But  is true at *w*_2_, and  is not, showing that (b) does not entail (c).

36 It is not clear to us whether Williamson makes a distinction between supposing and imagining as mental activities. In the quotes provided below, he speaks of supposing. In his book he also speaks, however, of imagination as essentially involved in our evaluation of counterfactuals. And we note that, for him (p. 170), “Imagine that there is a barber who shaves all and only those who do not shave themselves” is not radically different from the instruction “Suppose that there is a barber who shaves all and only those who do not shave themselves”. On how imagining may differ from supposing, see Balcerak Jackson [1].
